# Inactivation of Glucocorticoid Receptor in Noradrenergic System Influences Anxiety- and Depressive-Like Behavior in Mice

**DOI:** 10.1371/journal.pone.0072632

**Published:** 2013-08-20

**Authors:** Piotr Chmielarz, Justyna Kuśmierczyk, Rosanna Parlato, Günther Schütz, Irena Nalepa, Grzegorz Kreiner

**Affiliations:** 1 Department of Brain Biochemistry, Institute of Pharmacology, Polish Academy of Sciences, Cracow, Poland; 2 Department of Molecular Biology of the Cell I, DKFZ-ZMBH Alliance, German Cancer Research Center, Heidelberg, Germany; 3 Institute of Anatomy and Cell Biology, University of Heidelberg, Heidelberg, Germany; 4 Institute of Applied Physiology, University of Ulm, Ulm, Germany; Université Pierre et Marie Curie, France

## Abstract

The aim of this study was to investigate whether conditional inactivation of the glucocorticoid receptors (GRs) in noradrenergic neurons affects animal behavior in mice. Selective ablation of GRs in the noradrenergic system was achieved using the Cre/loxP approach. We crossed transgenic mice expressing the Cre recombinase under the dopamine beta-hydroxylase (DBH) promoter with animals harboring the floxed GR gene. The resulting GR^DBHCre^ mutant mice exhibited no alterations in terms of normal cage behavior, weight gain, spatial memory or spontaneous locomotor activity, regardless of gender. To assess depressive- and anxiety-like behaviors we performed the Tail Suspension Test and the Light-Dark Box Test. While male mutant animals did not show any alternations in both tests, female GR^DBHCre^ mutants displayed depressive- and anxiety-like behavior. Additionally, male GR^DBHCre^ mice were exposed to chronic restraint stress but still exhibited immobility times and anxiety statuses similar to those of non-stressed animals while stressed control mice clearly revealed depressive- and anxiety-like phenotype. Thus, in males the effects of the mutation were precipitated only after chronic restraint stress procedure. Our data reveal a possible gender-dependent role of GRs in the noradrenergic system in anxiety- and depressive-like behavior in mice.

## Introduction

Noradrenergic system and hypothalamic-pituitary-adrenals (HPA) axis are two major systems involved in stress response. Stress triggers many physiological and behavioral responses to maintain homeostasis in the organism. However, if the stress response is sustained, it may produce a vulnerable phenotype resulting in various health problems [1]. Acute stress exposure activates the brain noradrenergic system, which is responsible for promoting immediate responses to perceived threats, e.g., by facilitating sensorimotor reflexes [2], modulating attention [3] and promoting anxiety-like behavior [4]. Furthermore, increasing noradrenaline (NA) levels promotes active escape behaviors (e.g., struggling and climbing) in a Forced Swimming Test (FST) [5]. In contrast, HPA axis is mainly responsible for long term stress adaptation [1]. The noradrenergic system {Kitada, 1983 #316}modulates the stress response mainly through its action on the limbic system and mobilization of body reserves through the activation of the sympathetic nervous system and promotion of adrenaline release from adrenal medulla [6]. Noradrenergic neurons may also be involved in the stimulation of the HPA axis. This action can be either direct, thorough innervations of the hypothalamic paraventricular nucleus [7], or indirect, through the influence of noradrenaline on limbic structures, which, in turn, activate the HPA axis themselves [6].

Stress-induced hyperactivity of the HPA system is believed to be a major contributor to the pathology of depression [8]. The activity of the HPA is controlled by glucocorticoid receptors (GRs), and the function of these receptors may be impaired in depression, resulting in reduced GR-mediated negative feedback on the HPA axis. Indeed, mice carrying GR mutations exhibit alterations in the HPA comparable to those observed in depressed patients [9]. Although classical homozygous GR knockout mice are not available due to their lethality [10], GR under- (heterozygous GR^+/-^) and over-expressing (YGR) mice display stress-induced depressive-like and anti-depressive phenotypes, respectively [9,11].

The aim of the current study was to investigate whether conditional inactivation of GRs in the noradrenergic neurons of mice affects the animals’ behavior and whether this effect is similarly expressed in both genders.

## Materials and Methods

### Animals

All tested animals were of the C57BL/6N strain. Selective ablation of GRs in the noradrenergic system (GR^DBHCre^ mice) was achieved using the Cre/loxP approach. Transgenic mice hosting Cre recombinase under the dopamine beta-hydroxylase (DBH) promoter were crossed with animals harboring the floxed GR gene as described previously [12]. Previous studies performed on GR^DBHCre^ mice revealed the crucial role of GRs in postnatal maintenance of chromaffin cells, resulting in the inhibition of adrenaline synthesis [13]. Male and female mutant mice were kept with their control (Cre-negative) littermates of the same sex in self-ventilated cages under standard laboratory conditions (12 h light/dark cycle, food and water *ad libitum*). Animals were 12 weeks old at the time of the behavioral tests.

This study was carried out in strict accordance with the recommendations in the Guide for the Care and Use of Laboratory Animals of the National Institutes of Health. The protocol for all the behavioral study was approved by the Animal Ethical Committee at the Institute of Pharmacology, Polish Academy of Sciences (Permit Number: 789, issued: Sept 30, 2010).

### Behavioral experiments

*Open Field Test (OFT)* was used to measure spontaneous locomotor activity. Mice were video recorded for 60 minutes in 40x40 cm square boxes, and the total distance moved was scored in 10 min intervals.

*Elevated Plus Maze (EPM)* was utilized to assess short term-spatial memory as described by [14]. Briefly, a mouse was placed on the open arm of a dimly illuminated (30 lux) EPM facing outwards, and the latency of entry into the closed arm was measured. Spatial memory was assessed by repeating the procedure 24 hours later, when decreased latency to enter closed arm serves as index of spatial memory function as shown by [15] [14] [16].

*Tail Suspension Test (TST)* was utilized to assess depressive-like behavior; the time the animals were immobile while suspended by the tail for 6 minutes was taken as a measure of depressive-like behavior. Scoring of immobility time was performed by means of automated video tracking software EhtoVision XT8 (Noldus), utilizing similar method to that described by [17]. Animal shape was detected automatically by software using greyscaling method basing on high contrast between black animal and white background. Mobility of animal is assessed by comparing detected animal shape in frame *n* with that in frame *n-1*, result presented as percentage of area change. Video tracked records were acquired at 25 frames/second and threshold for scoring immobility was set to 5% area change averaged over 1s intervals. *Light-Dark Box* (*LD Box*) was performed according to the guidelines provided in [18]. Male animals were put into the light part of a box consisting of connected light (400 lux) and dark (40 lux) compartments, and the time spent in the light compartment over a 5 minute trial was measured.

*Chronic restraint stress* procedure was performed by placing animals, for 2 hours daily, in 50 ml disposable centrifuge tubes that were adapted for this purpose by drilling holes to permit air circulation. This procedure was repeated for 14 consecutive days, and behavioral experiments were performed 24 hours after the last restraint stress. Animals where weighted before first day of experiment and at the end of 1^st^ and 2^nd^ week of the procedure.

All the experiments were performed by experimenters blind to animal genotypes, video tracked and scoring of behavioral experiments was performed automatically or semi-automatically by means of automated video tracking software EhtoVision XT8 (Noldus). To ensure the accuracy of automated analysis the detection settings for the TST test were validated by comparing time course of immobility scored manually with that scored by the software in selected animals (Figure S1). This observation was is in line with other authors who also compared the data obtained manually and automatically with use of mentioned above software [17].

### Corticosterone analysis

Blood samples were taken 1 hour after the start of the light phase of the light/dark cycle, either immediately (basal level) or 30 min after a half hour of immobilization stress (performed as described above). Blood samples (3-4 drops/sample) were rapidly collected from decapitated animals and plasma corticosterone levels were determined with use of the Corticosterone ELISA kit (DRG instruments, Germany) according to the manufacturer’s instructions.

### Tissue preparation and processing

Animals were sacrificed by cervical dislocation, and their brains were removed and fixed in 4% paraformaldehyde overnight, embedded in paraffin and sectioned on a rotary microtome, for coronal sections (7 µM) for different brain areas (hippocampus, locus caeruleus).

### Immunofluorescence staining

Chosen sections from corresponding regions in mutant and control animals were incubated overnight at 4 °C with primary anti-GR (1:50, Abcam, Cambridge, UK) and anti-TH (1:1000, Millipore) antibodies. Antigen-bound primary antibodies were visualized with anti-rabbit Alexa-488 and anti-goat Alexa-594 coupled secondary antibodies.

## Results and Discussion

**Figure 1:**
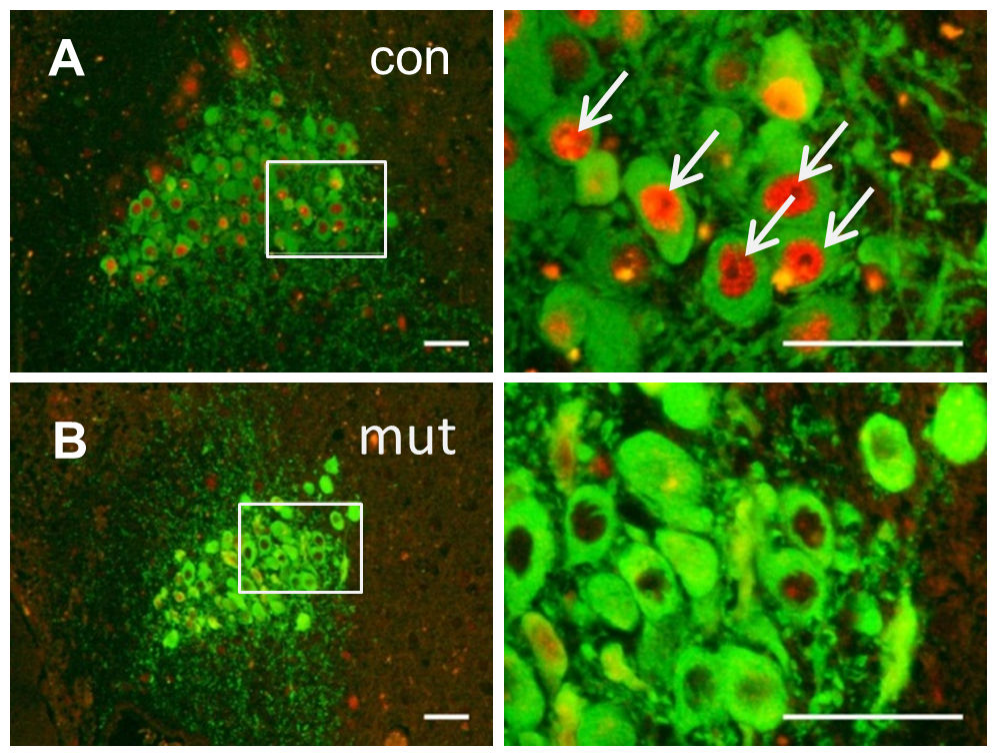
Lack of glucocorticoid receptor (GR) expression in the region of locus caeruleus in mutant mice. Superimposed immunofluorescence images of glucocorticoid receptor (GR, red) and tyrosine hydroxylase (TH, green) in locus coeruelus of control (A) and mutant (B) animals. White arrows show clear GR staining in TH positive (TH+) cells of control animal, the staining is absent in the TH+ cells of mutant. Scale bars: 50 µm for left side and 25 µm for right side images.

The specificity of introduced mutation was previously validated only at peripheral tissues as described before [13]. Thus, in the first experiment, we validated the specificity of the mutation within the central nervous system as well. As expected, GR expression was selectively and completely lost in the region of the locus coeruleus (LC) (Figure 1), but not in other brain structures like hippocampus (Figure S2).

**Figure 2:**
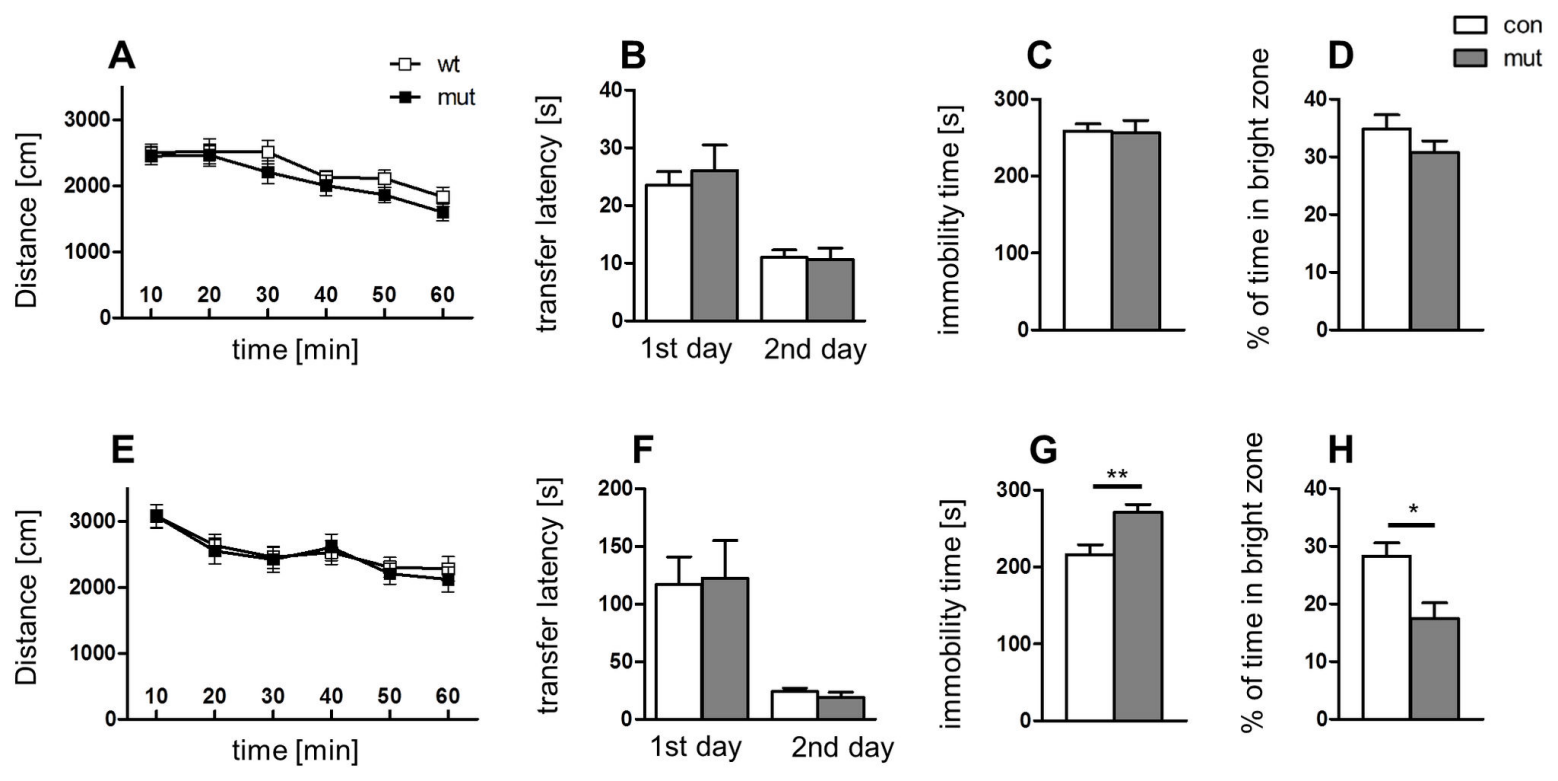
Behavioral comparison of control (con) and mutant (mut) male (A–D) and female (E–H) mice. **A, E** – locomotor activity assessed by mean distance traveled in open field during 10 min intervals; **B, F** – spatial memory after 24h evaluated by transfer latency in elevated plus maze. **C, G** depressive-like behavior reflected by immobility time assessed by Tail Suspension Test (TST), **D, H** - anxiety-like behavior measured by percentage of time.

**Figure 3:**
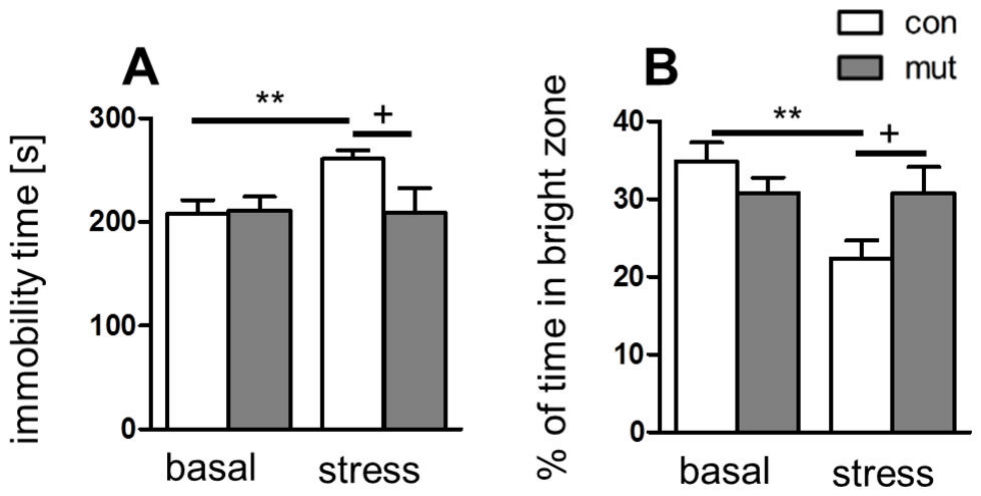
Chronic stress effects on anxiety and depressive-like behavior of control (con) and mutant (mut) males. **A** – depressive-like behavior reflected by immobility time assessed by Tail Suspension Test (TST); **B** – anxiety-like behavior measured by percentage of time spent in bright zone in Light-Dark Box Test (LDT), Values represent means ± SEM, n=6-10 mice. **p<0.01 vs non-stressed (basal) control animals, +p<0.05 vs stressed control animals.

Next, we performed a series of behavioral experiments to further characterize the phenotype of GR^DBHCre^ mutant mice. Because a growing number of studies have indicated that gender differences may influence HPA axis activity and depressive-like symptomatology in behavioral tests [19], all experiments in unstressed animals were conducted in parallel on male and female cohorts. We did not observe any differences in spontaneous locomotor activity, spatial memory in male (Figure 2A,B) nor female mutant mice (Figure 2E,F). When compared to control animals, GR^DBHCre^ mice displayed no obvious alterations in daily cage behavior nor weight gain (Figure S3). Overall, the GR^DBHCre^ mice did not seem to be retarded in terms of basic behavior, despite the complete, selective ablation of GRs in the LC and the degeneration of chromaffin cells leading to loss of adrenaline [13]. To assess whether the GR^DBHCre^ mice may be affected in terms of depressive- and anxiety-like behavior, Tail Suspension Test (TST) and Light-Dark Box Test (LDT) were performed. Interestingly, female mutant mice displayed clear depressive-like behavior (25% increase in immobility time in the TST; p<0.01) (Figure 2G) and an anxiety-like phenotype (increased latency to enter the light compartment in the LDT; mutant = 90 s vs. control = 33 s; p<0.01) (Figure 2H) in the basal, non-stressed state, while no changes in these parameters were observed in male mutant mice (Figure 2C,D). Because male mutants did not exhibit any alternations under basal state and mutation targets systems are involved in long term stress adaptation we decided to test whether chronic restraint stress might precipitate expression of phenotype in these mice. Chronic stress may significantly alter the function of the noradrenergic system [20] and be an important etiological factor in mood and anxiety disorders [21,22]. The involvement of chronic stress in these pathologies is reflected by the facilitatory effects of chronic stress models on the depressive- and anxiety-like behaviors of laboratory animals, although the effects on the latter are not always conclusive [23]. Thus, we tested whether chronic stress could evoke the depressive- and anxiety-like symptoms in GR^DBHCre^ male mice by comparing both non-stressed and chronically stressed animals using the Tail Suspension Test and the Light-Dark Box Test. To ensure that utilized procedure of restraint stress was really stressful for mice, their weights were constantly monitored and revealed similar weight loss in both control and mutant animals over the 2 weeks (Figure S4). Chronic stress indeed precipitated behavioral differences in mutant and control males, however in unexpected manner - both TST and LDT tests revealed that male GR^DBHCre^ mutants (Figure 3A,B) were resistant to this type procedure. Stressed and non-stressed mutant males exhibited similar immobility times and anxiety statuses like non-stressed control animals. As expected, chronically stressed control males showed increased immobility time in the TST (25% increase, p<0.01) and decreased time spent in the light compartment in the LDT (by 35%, p<0.01), which response is in line with other studies regarding chronic stress effects in depression and anxiety tests [24].

Loss of GR in noradrenergic neurons does not lead to changes of basal behavior and renders male GR^DBHCre^ mutants more resistant to chronic stress than control littermates. Our findings corroborate studies reporting that variations in glucocorticoid levels may not influence the noradrenergic system directly but rather by modulating the changes caused by chronic stress exposure [25]. Thus, our results suggest that it is specifically the activation of GRs in noradrenergic neurons which activation during chronic stress is important for the modulation of the noradrenergic system and what in turn leads to alterations in anxiety- and depressive-like behaviors. This hypothesis would correspond to the results obtained from mice lacking the noradrenergic transporter (NET-/-). Similarly to the GR^DBHCre^ males, these mutant mice display stress-related weight loss after chronic stress exposure (although the nature of the stressor was different than that in the present study) but do not display enhanced depressive-like behavior in the forced swim test [26]. However, we cannot rule out the possibility that differential modulation of the noradrenergic system by chronic stress in GR^DBHCre‑^ animals results, at least partially, from abnormal peripheral adrenaline levels; the mechanisms of such chronic modulation could also be investigated in our animals.

Nevertheless, it remains interesting that female GR^DBHCre^ mutants, unlike males, displayed increased anxiety- and depressive-like behaviors in the basal state. This gender dependent difference in response to mutation was additionally confirmed by 2-way ANOVA test which revealed gender*genotype (p<0.05) interaction in TST, altought gender*genotype interaction in LDT test was statistically insignificant.

Neurochemical and molecular investigation should help to better understand these unexpected results. However, we can speculate that higher circulating glucocorticoid levels in females are important for maintaining the normal function of noradrenergic neurons, in contrast to males, and that this function is compromised by the deletion of GRs. The influence of the impairment of adrenal medulla function is unlikely to be the cause of the behavior observed here because similar changes should have been observed in other behavioral tests, particularly the OFT. Interestingly, it has previously been reported that the LDT but not the OFT is responsive to noradrenergic manipulation by selective NA reuptake inhibitors [27] and conversely phenotype of GR^DBHCre^ female mutants expressing in LDT but not OFT could suggest abnormal noradrenergic function.

Furthermore, the HPA axis can influence noradrenergic systems in several ways. First, glucocorticoids can modulate both the synthesis and reuptake of NA by modulating the expression of both tyrosine hydroxylase (TH, the rate-limiting enzyme in the synthesis of NA) [25] and the NA transporter (NET) [28]. These modulations could possibly influence basal levels of NA and tonic sympathetic nervous system activity, but glucocorticoids can also modulate the availability of NA by regulating α_2_-adrenergic receptor-mediated inhibition of NA release [29]. Thus, it is conceivable that GRs in noradrenergic neurons might themselves play a role in the reported changes in their reactivity after chronic stress exposure [20]. Through such effects on the noradrenergic system, GRs may influence animal behavior, as the noradrenergic system is widely implicated in mood and anxiety disorders – conditions that are often related to excessive (real or perceived) stress exposure and HPA axis dysfunction.

**Figure 4:**
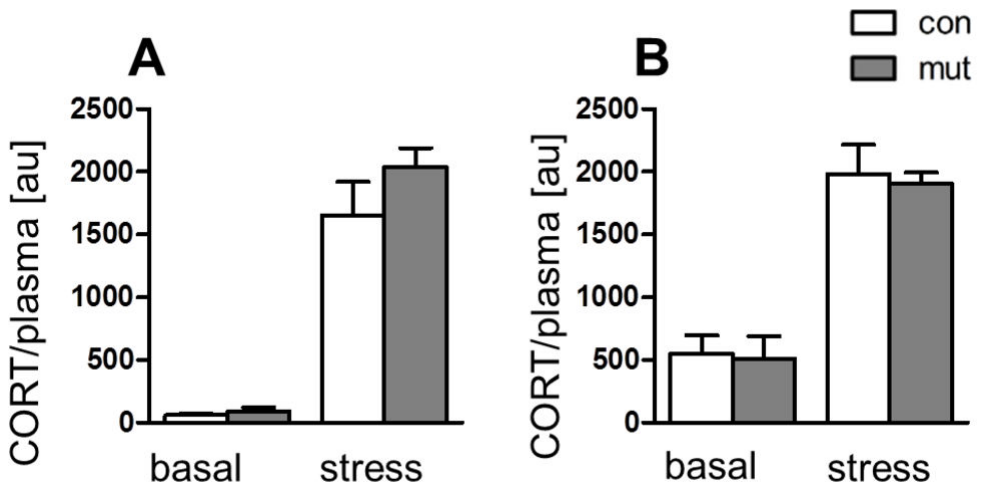
Plasma corticosterone levels. Assessment of plasma corticosterone levels of male (**A**) and female (**B**) control (con) and mutant (mut) mice in basal state and 30 min after chronic restraint stress.

To establish whether the observed gender-dependence is linked to the differential responses of the HPA axis to stressful stimuli, we measured plasma levels of corticosterone under basal and stressed conditions in both control and mutant animals. No differences were observed in plasma corticosterone levels under basal or stress conditions in control and mutant animals, whether male or female (Figure 4A,B). This observation suggests that the gender-dependent response to the mutation and observed resistance to stress in male GR^DBHCre^ mutants cannot be explained simply by any alterations in HPA axis. However, the higher levels of basal plasma corticosterone in female animals may influence the depressive-like behavior observed in non-stressed female mutants. Women are considered to be more prone to depression than men and this vulnerability is reflected in animal models [30]. Thus, one can speculate that GR^DBHCre^ female mutants might have lower stress tolerance thresholds.

Our data unravel a possible gender-dependent role of GRs in the noradrenergic system in anxiety- and depressive-like behavior in mice and indicate the GRs dependent regulation of restraint stress response in males. Further research is needed to dissect the possible molecular mechanisms mediating this phenomenon.

## Supporting Information

Figure S1 **Comparison of automatic and manual scoring results of tail suspension test obtained with use of EhtoVision XT8.**
(TIF)

Figure S2 **Images from hippocampal regions of Dentate Gyrus (A, D), CA1 (B, E) and CA3 (C, F).**
Images show similar pattern of GR staining in control (**A**–**C**) and mutant (**D**–**F**) mice. Scale bars: 100 µm.
(TIF)

Figure S3 **Weight of male (A) and female (B) animals at different age.**
Both control and mutant mice show similar weight gain. n = 12.
(TIF)

Figure S4 **Weight change of control and mutant male animals during procedure of chronic restraint stress measured at the end of 1st and 2nd week of the procedure.**
(TIF)
