# Supplementary figures and images for: Inactivation of Glucocorticoid Receptor in Noradrenergic System Influences Anxiety- and Depressive-Like Behavior in Mice
Source: PLoS One. 2013 Aug 20;8(8):e72632. doi: 10.1371/journal.pone.0072632 (PMC3748181; doi:10.1371/journal.pone.0072632)

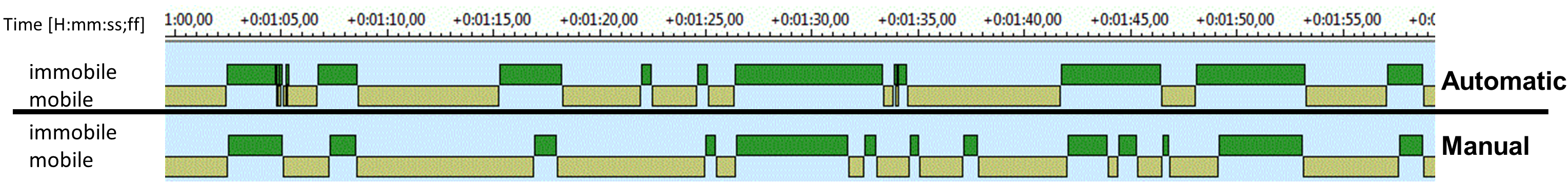

Supplement: Figure S1 — Comparison of automatic and manual scoring results of tail suspension test obtained with use of EhtoVision XT8. (TIF) [file pone.0072632.s001.tif]

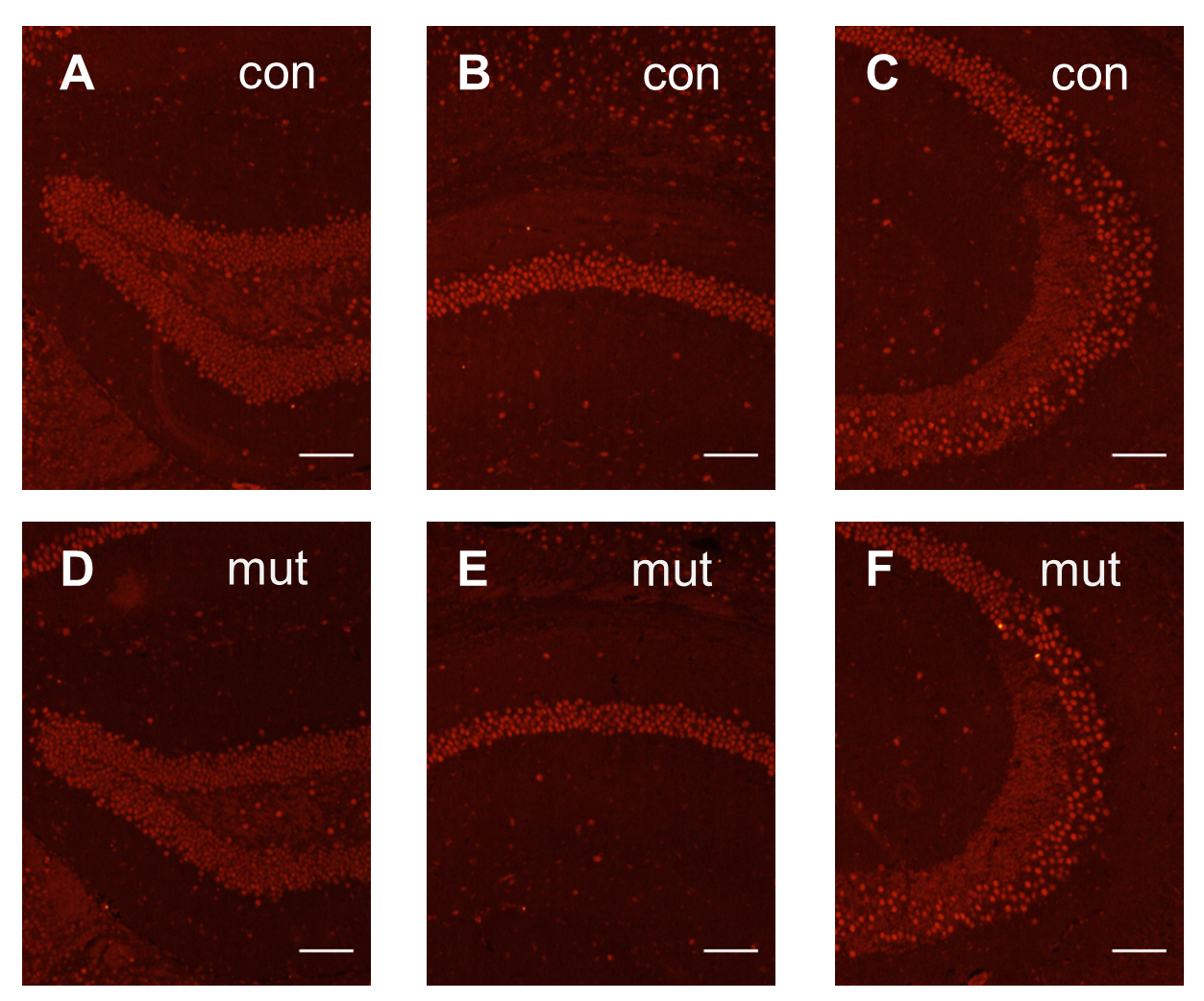

Supplement: Figure S2 — Images from hippocampal regions of Dentate Gyrus (A, D), CA1 (B, E) and CA3 (C, F). Images show similar pattern of GR staining in control (A–C) and mutant (D–F) mice. Scale bars: 100 µm. (TIF) [file pone.0072632.s002.tif]

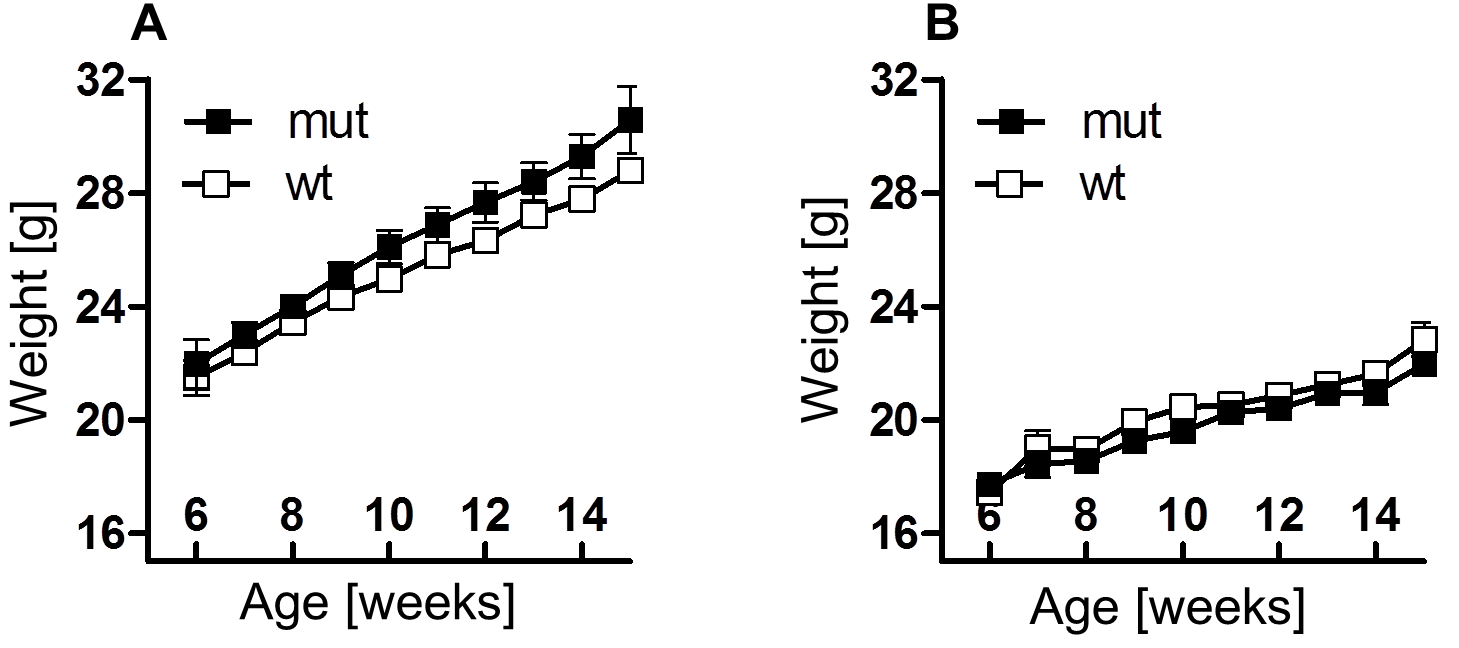

Supplement: Figure S3 — Weight of male (A) and female (B) animals at different age. Both control and mutant mice show similar weight gain. n = 12. (TIF) [file pone.0072632.s003.tif]

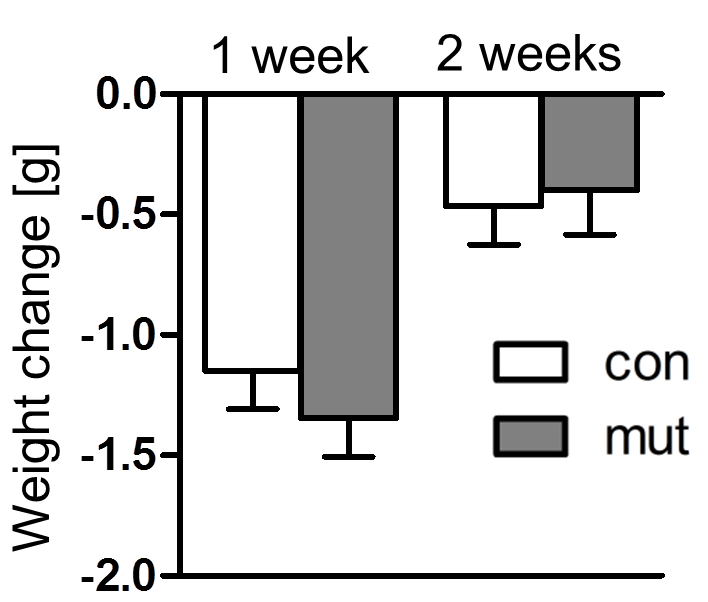

Supplement: Figure S4 — Weight change of control and mutant male animals during procedure of chronic restraint stress measured at the end of 1st and 2nd week of the procedure. (TIF) [file pone.0072632.s004.tif]
